# Acupuncture and Clomiphene Citrate for Live Birth in Polycystic Ovary Syndrome: Study Design of a Randomized Controlled Trial

**DOI:** 10.1155/2013/527303

**Published:** 2013-08-20

**Authors:** Hongying Kuang, Yan Li, Xiaoke Wu, Lihui Hou, Taixiang Wu, Jianping Liu, Ernest Hung Yu Ng, Elisabet Stener-Victorin, Richard S. Legro, Heping Zhang

**Affiliations:** ^1^Department of Obstetrics and Gynecology, First Affiliated Hospital, Heilongjiang University of Chinese Medicine, Harbin 150040, China; ^2^Chinese Clinical Registry, Chinese Cochrane Centre, Chinese Evidence-Based Medicine Centre, INCLEN CERTC, West China Hospital, Sichuan University, Chengdu 610041, China; ^3^Centre for Evidence-Based Chinese Medicine, Beijing University of Chinese Medicine, Bei San Huan Dong Lu 11, Chaoyang District, Beijing 100029, China; ^4^Department of Obstetrics and Gynecology, The University of Hong Kong, Queen Mary Hospital, Hong Kong Special Administrative Region, China; ^5^Institute of Neuroscience and Physiology, Department of Physiology, Sahlgrenska Academy, University of Gothenburg, 405 30 Gothenburg, Sweden; ^6^Department of Obstetrics and Gynecology, Pennsylvania State University College of Medicine, Hershey, PA 17033, USA; ^7^Department of Biostatistics, Yale University School of Public Health, New Haven, CT 06511, USA

## Abstract

Acupuncture is an alternative therapy to induce ovulation in women with polycystic ovary syndrome (PCOS), but there is no study reporting the live birth rate following ovulation induction by acupuncture or its potential as an adjuvant treatment to clomiphene citrate (CC). We assess the efficacy of acupuncture with or without CC in achieving live births among 1000 PCOS women in Mainland China. This paper reports the methodology of an ongoing multicenter randomized controlled trial. The randomization scheme is coordinated through the central mechanism and stratified by the participating sites. Participants will be randomized into one of the four treatment arms: (A) true acupuncture and CC, (B) control acupuncture and CC, (C) true acupuncture and placebo CC, and (D) control acupuncture and placebo CC. To ensure the quality and integrity of the trial we have developed a unique multinational team of investigators and Data and Safety Monitoring Board. Up to the end of April 2013, 326 subjects were recruited. In conclusion, the success of this trial will allow us to evaluate the additional benefit of acupuncture beyond the first line medicine for fertility treatment in PCOS women in an unbiased manner.

## 1. Introduction

Polycystic ovary syndrome (PCOS) is the most common endocrine disorder in women of reproductive age. It is characterized by ovulatory dysfunction, hyperandrogenism, and polycystic ovaries (PCO) and affects 5–10% of premenopausal women [1]. In addition to irregular menses, hirsutism, and infertility, women with PCOS may display a number of metabolic abnormalities including hyperinsulinemia, insulin resistance, dyslipidemia, and obesity [2]. Clomiphene citrate (CC) is considered as the first line treatment to induce ovulation in women with PCOS. A compilation of published results from 5,268 patients revealed an ovulation rate of 73% per cycle per patient, pregnancy rate of 36% per patient, and live birth rate of 29% per patient during a six-month treatment [3]. Women who do not ovulate while receiving the maximum 150 mg dose are considered to be CC resistant and those who do not conceive after an ovulatory response are considered clomiphene failures. Both appear to be common [4], necessitating the search for adjuvant therapies to treat infertility in women with PCOS. 

Inability of CC to induce ovulation is more likely in patients who are obese, insulin resistant, and hyperandrogenic compared with those who do respond [5]. The mechanism of CC failures may partly be explained by a peripheral anti-estrogenic effect at the level of endometrium and cervical mucus or by hypersecretion of luteinizing hormone (LH). Side effects of CC are related to its combined estrogenic and antiestrogenic properties, which include hot flushes, breast discomfort, abdominal distension, nausea, vomiting, nervousness, sleeplessness, headache, mood swings, dizziness, hair loss, and disturbed vision [4]. Approximately 7% of pregnancies resulting from CC-induced ovulation are twin pregnancies and 0.5% are triplet pregnancies [6]. 

The choice of adjuvant treatments or second line treatment is controversial. The benefit of combined metformin/CC has been called into question by the meta-analysis [7]. Gonadotropin treatment can be offered as the second line drug when anovulatory PCOS women fail to respond to CC. The use of gonadotropin is more expensive than CC and associated with a higher risk of multiple pregnancies and development of ovarian hyperstimulation syndrome. Ovarian drilling by diathermy or other methods is the second line therapy with equal efficacy to gonadotropin [8]. However this treatment is also expensive and invasive. New or additional treatments that are readily accessible, affordable, and safe are needed.

Acupuncture is an integral part of Traditional Chinese Medicine (TCM), which dates back to more than 3,000 years. In recent years, the use of acupuncture within reproductive endocrinology and infertility has gained increased popularity worldwide [9–11]. From a physiological perspective, the effect of acupuncture is most likely mediated via activation of sensory nerve fibers, which in turn modulate the sympathetic activity to the ovaries and from the central nervous system (CNS). Support for the theory that acupuncture, at least in part, modulates sympathetic activity arises from the findings that acupuncture decreases high ovarian expression of sympathetic markers and increases ovarian blood flow, an effect mediated as a reflex response via ovarian sympathetic nerves and via CNS [12–14]. Further, both acupuncture and exercise reduced high muscle sympathetic nerve activity in women with PCOS [15, 16].

In PCOS women with ovulatory dysfunction, several nonrandomized trials revealed that repeated acupuncture treatments decreased total testosterone and other sex steroid levels, reduced LH/follicle-stimulating hormone (FSH) ratio, and improved menstrual frequency without negative side effects [17–19]. In a randomized controlled trial (RCT), fourteen acupuncture treatments, with combined electrical and manual stimulation of the needles during 16 weeks and 16 weeks of physical exercise compared with no intervention, improved menstrual bleeding pattern and decreased high levels of sex steroid precursors, estrogens, androgens, and glucuronidate androgen metabolites in women with PCOS [20]. Acupuncture was superior to physical exercise when compared directly after the treatment period. In a quasi-randomized study, daily abdominal acupuncture during 6 months improved menstrual frequency and decreased circulating testosterone more effectively than metformin during 6 months [21]. Recently in another RCT, 12 treatments of true acupuncture were compared with control acupuncture using placebo needles during 8 weeks and similar ovulation rates were found in both groups of women with PCOS [22]. 

Findings from previous clinical and experimental studies indicate that acupuncture may induce ovulation and improve pregnancy in women with PCOS. However, the methodological quality of these studies is of varying quality, and none were designed to investigate pregnancy or more importantly live birth. We sought to address these gaps in knowledge by designing an RCT to examine the effects of acupuncture on live birth in women with PCOS. The objective of the present trial is to test the following three hypotheses in anovulatory women with PCOS: (1) true acupuncture and CC are more likely to result in live birth than control acupuncture and CC, (2) control acupuncture and CC are more likely to result in live birth than true acupuncture and placebo CC, and (3) true acupuncture and placebo are more likely to result in live birth than control acupuncture and placebo CC.

## 2. Materials and Methods

### 2.1. Study Population

Subjects will be recruited from 25 hospitals across 13 provinces in Mainland China when they seek treatments for ovulatory problems and pregnancies. They will be eligible if they meet the following inclusion and exclusion criteria. Informed consent will be obtained after detailed explanation, and each will be given a copy of the signed consent.

Inclusion criteria are as follows.

1. Age of women between 20 and 40 years.
2. Chronic oligomenorrhea or amenorrhea: oligomenorrhea is defined as an intermenstrual interval >35 days or <8 menstrual bleedings in the past year. Amenorrhea is defined as an intermenstrual interval >90 days.
3. Hyperandrogenism (either hirsutism or hyperandrogenemia) or polycystic ovaries on ultrasound. Hirsutism is determined by a modified Ferriman-Gallwey Score ≥ 5 at screening examination [23]. Polycystic ovaries are present when there are ≥12 antral follicles (2–9 mm) or ovarian volume >10 mL on transvaginal scanning [24].
4. Further, at least one patent tube and normal uterine cavity shown by hysterosalpingogram, HyCoSy, or diagnostic laparoscopy with chromotubation within three years [25].
5. Sperm concentration ⩾15 × 10^6^/mL and total motility ⩾ 40% or total motile sperm count ⩾  10 million in the semen analysis of the husband [26, 27].
6. Agree to have regular intercourse, that is, 2-3 times per week during the study period.

Exclusion criteria are as follows.

1. Exclusion of other endocrine disorders: *①* patients with hyperprolactinemia ( defined as two prolactin levels at least one week apart ⩾25 ng/mL) *②* Patients with FSH levels > 15 mIU/mL. A normal level within the last year is adequate for entry. *③* Patients with uncorrected thyroid disease (defined as TSH <0.2 mIU/mL or >5.5 mIU/mL). A normal level within the last year is adequate for entry. *④* Patients diagnosed with Type I or Type II diabetes who are poorly controlled (defined as HbA1c level > 7.0%), or patients receiving antidiabetic medications such as metformin, insulin, thiazolidinediones, acarbose, or sulfonylureas. *⑤* Patients with suspected Cushing's syndrome.
2. Use of hormonal or other medication including Chinese herbal prescriptions in the past 3 months.
3. Pregnancy within the past 6 weeks.
4. Within 6 weeks postabortion or postpartum.
5. Breastfeeding within the last 6 months.
6. Not willing to give written consent to the study.
7. Additional exclusion criteria are as follows.
  - Patients on oral contraceptives, depot progestins, or hormonal implants (including Implanon). A two-month washout period will be required prior to screening for patients on these agents. Longer washouts may be necessary for certain depot contraceptive forms or implants, especially where the implants are still in place. A one-month washout will be required for patients on oral cyclic progestins.
  - Patients with liver disease defined as AST or ALT > 2 times normal or total bilirubin > 2.5 mg/dL. Patients with renal disease defined as BUN > 30 mg/dL or serum creatinine > 1.4 mg/dL.
  - Patients with hemoglobin < 10 g/dL.
  - Patients with a history of deep venous thrombosis, pulmonary embolus, or cerebrovascular accident.
  - Patients with known heart disease that is likely to be exacerbated by pregnancy.
  - Patients with a history of, or suspected cervical carcinoma, endometrial carcinoma, or breast carcinoma. A normal Pap smear result will be required for women 21 and over.
  - Patients with a current history of alcohol abuse. Alcohol abuse is defined as >14 drinks/week or binge drinking.
  - Patients enrolled into other investigative studies that require medications, prescribe the study medications, limit intercourse, or otherwise prevent compliance with the protocol.
  - Patients who anticipate taking longer than a one-month break during the protocol should not be enrolled.
  - Patients taking other medications known to affect reproductive function or metabolism. These medications include oral contraceptives, GnRH agonists and antagonists, antiandrogens, gonadotropins, antiobesity drugs, Chinese herbal formula, antidiabeteic drugs such as metformin and thiazolidinediones, somatostatin, diazoxide, angiotensin-converting-enzyme (ACE) inhibitors, and calcium channel blockers. The washout period on all these medications will be two months.
  - Patients with a suspected adrenal or ovarian tumor secreting androgens.
  - Couples with previous sterilization procedures (vasectomy, tubal ligation), which have been reversed. The prior procedure may affect study outcomes, and patients with both a reversed sterilization procedure and PCOS are rare enough that exclusion should not adversely affect recruitment.
  - Subjects who have undergone a bariatric surgery procedure in the recent past (<12 months) and are in a period of acute weight loss or have been advised against pregnancy by their bariatric surgeon.
  - Patients with untreated poorly controlled hypertension defined as a systolic blood pressure 160 mm Hg or a diastolic 100 mm Hg obtained on two measures obtained at least 60 minutes apart.
  - Patients with known congenital adrenal hyperplasia.

### 2.2. Study Design

This is a multicenter, randomized, double-blinded, controlled, 2 by 2 factorial clinical trial for PCOS women in China, with study treatment during 4-month cycles. Participants are randomized to receive either an initial oral dose of 50 mg of CC or placebo CC from day 3 to day 7 of the cycle [4] in combination with one of the two acupuncture procedures. The starting date for CC is not expected to affect the treatment outcome [28]. In order to maintain the double-blinding for the medication, CC and placebo CC were packaged in identically appearing numbered study kits which were then directly shipped to each clinical site. For acupuncture, all patients will receive either true or control acupuncture treatment. 

### 2.3. Randomization

Randomization takes place as soon as the eligibility requirements are met. Participants are randomized into one of the four treatment arms: (1) true acupuncture and CC, (2) control acupuncture and CC, (3) true acupuncture and placebo CC, and (4) control acupuncture and placebo CC by an interactive online computer program in a central office. Using a 1 : 1 : 1 : 1 treatment ratio, there will be 250 women assigned to each treatment group and we expect the dropout rate would be 20%. The randomization is stratified within each of the participating sites by a random block algorithm. The data coordinating center (DCC) statisticians generated and validated the randomization scheme for the study before it was implemented in a web-based database system, and the study coordinator at each participating site logs on the web-based system to retrieve the randomization number after a patient is ready for it. The CC and placebo CC assignments are double-blinded, unknown to patients and study investigators except the DCC data manager. The true and control acupuncture treatments are known only to the TCM specialist (besides the DCC data manager) of the participating site that performs the randomized procedure. 

### 2.4. Acupuncture Treatments

Two fixed acupuncture protocols, true acupuncture and control acupuncture, are used in the study. The rationale of true acupuncture is based on existing data from experimental studies and on previous RCT in women with PCOS [20, 22]. The control acupuncture is chosen in contrast to true acupuncture and designed in such a way that it is thought to be less effective for ovulation induction. The two acupuncture protocols follow the STRICTA recommendations [29]. 

Clinical experience and training of acupuncturists may affect the outcome [30]. To assure that the intervention is performed in a standard manner, all acupuncturists with TCM certificate and 1 year experience receive a study specific theoretical course and a study specific practical training, totally 3 times before the beginning of the study, 1–3 days per time, and 1 time, four hours after one month of the study beginning. All acupuncturists have to pass the theory and practical tests. In China, acupuncturists perform acupuncture in over 50 patients per day, thus they will rapidly be experienced. Furthermore, while they are trained for only a short time for this study acupuncture protocols, they are graduate students in Chinese medicine and have received general acupuncture training during their studies. In this study, 35 acupuncturists have been trained both in theory and practice of acupuncture, and there is at least one designated acupuncturist at each participating site. 

All participants receive acupuncture treatment twice a week and each treatment session can be separated by an interval of 2–4 days, with a maximum of 32 treatment sessions during 16 weeks. Each treatment session lasts for 30 minutes. Acupuncture treatment will start on day 3 after a spontaneous period or a withdrawal bleeding following progestin, when starting with CC/placebo tablet. Participants will be contacted by phone if they miss the appointment as scheduled. If they miss appointments in the early part of the cycle, more appointments will be arranged in the later part to compensate the missing part. Missed appointments will be clearly documented in the record for analysis later. If the participants miss 6–8 acupuncture treatments (3-4 weeks), she should be scheduled not to take CC or CC placebo and have this treatment cycle “cancelled” until she can get acupuncture. 

In true acupuncture, needles are made of stainless steel (Hwoto, Suzhou Medical Appliance Fact., Suzhou, China); 0.25 × 30 mm and 0.30 × 40/50 mm needles are inserted to a depth of 15–35 mm in acupuncture points located in abdominal and leg muscles with same somatic innervations as the ovaries and uterus [31]. Two sets of acupuncture points alternated every other treatment. The first set of acupuncture points consists of conception vessel (CV) 3, CV 6, and stomach (ST) 29 bilaterally and in the muscles below the knee, spleen (SP) 6, and SP 9 bilaterally; all segmental points with somatic innervations correspond to the ovaries and the uterus (Table 1). Needles are also placed in extrasegmental acupuncture points that do not innervate the ovaries and uterus: large intestine (LI) 4 bilaterally and governor vessel (GV) 20. Abdominal and leg points were selected because they have been demonstrated to increase ovarian blood flow response [13, 14]. Points in hands and head have been used in previous studies [20] and were selected to potentiate the effect of the segmental points. Furthermore, they have been demonstrated in experimental studies to act via central nervous system [32]. 

A total of 11 needles are placed, and all are stimulated manually by rotating the needle quickly with thumb and forefinger to evoke needle sensation (de qi) once when inserted. De qi reflects activation of afferent nerve fibers and can be described as numbness, distension, or electrical tingling. The points CV 3, CV 6, ST 29, SP 6, and SP 9 are thereafter connected to electrical stimulator (AS Super 4 Digital, schwa-medico GmbH, Ehringshausen, Germany) and stimulated with low-frequency, 2 Hz, 0.3 ms pulse length and the intensity is adjusted to produce local muscle contractions without pain or discomfort. Needles not connected to the electrical stimulator are manually stimulated by rotating the needle to evoke needle sensation every 10 min, in a total of 4 times. If the sensation of de qi does not occur, manual manipulation techniques can be applied to promote it (such as “plucking,” “shaking,” or “trembling”). The second set of acupuncture points consists of 13 needles placed in segmental abdominal points; ST 25 and 29 bilaterally (electrical stimulation) and CV3 and CV6 (manual stimulation), leg points; SP6 and liver (LR) 3 bilaterally (electrical stimulation). Extrasegmental points are pericardium (PC) 6 bilaterally and GV20 (both manual stimulation). 

In control acupuncture, two needles will be inserted superficial to a depth of <5 mm in each arm, one in each shoulder and one in each upper arm at nonacupuncture points, without any stimulation (Table 1). Needles are made of stainless steel (Hwoto, Suzhou Medical Appliance Fact., Suzhou, China), 0.20 × 20 mm. Electrodes will be attached to the needles and the stimulator is turned on at an intensity of zero (no active current) in order to mimic electrical stimulation in the true acupuncture group. No stimulation of the needles is performed. 

Participants are monitored weekly by urinary pregnancy test and serum progesterone levels. The dose of oral medication may be increased by 50 mg in the absence of ovulation or maintained in the presence of ovulation. The maximum dose of CC will not exceed 150 mg/cycle. The treatment lasts for up to 4 month cycles. 

### 2.5. Study Specific Visits and Procedures

Each specific visit and measurement is summarized in Table 2. Baseline measures include fasting FSH, LH, total testosterone (T), estradiol (E2), sex hormone binding Globulin (SHBG), dehydroepiandrosterone sulfate (DHEAS), glucose and insulin concentrations, cholesterol, triglycerides, high-density lipoprotein cholesterol (HDL-C), low-density lipoprotein cholesterol (LDL-C) and height, weight, hip, waist measurements, and vital signs. Neither cervical mucus nor endometrial lining will be monitored in this large clinical trial as these are not routinely monitored in clinical practice. Also questionnaires for assessments of health related quality of life (QOL) are filled in: the short form 36 (SF 36) [33], the Polycystic Ovary Syndrome Questionnaire (PCOS-QOL) [34], and the Chinese Quality of life (ChQOL) [35]. Questionnaires for symptoms of anxiety and depression are Zung Self-Rating Anxiety Scale (SAS) [36] and Zung Self-reported Depression Scale (SDS) [37]. All questionnaires used in this trial are validated scales. 

The TCM diagnosis will be made by an experienced TCM doctor in each participating site according to a standard questionnaire. Syndrome differentiation in TCM is the comprehensive analysis of clinical information gained by the four main diagnostic TCM procedures: observation, listening, questioning, and pulse analysis [38]. In PCOS, patients are empirically differentiated to be four categories: (1) phlegm-dampness syndrome, (2) blood stasis syndrome, (3) phlegm and blood stasis, and (4) liver stagnation [39]. The TCM diagnosis will not affect the treatment protocol, and it will enable us to explore its effect on the treatment success in a secondary analysis.

All baseline measures will be repeated in all subjects at the end of visits. Blood samples (including the safety labs) will be collected and shipped to the core lab. Credibility check questionnaires are completed on the third acupuncture treatment and the last acupuncture treatment [40–42].

### 2.6. Responders

Responders are defined as having at least one weekly elevated serum progesterone level indicative of ovulation, that is, >3 ng/mL [43]. A woman who has ovulated will be followed weekly until she has a period or a positive pregnancy test. Responders will continue at the same dose of CC or placebo until pregnancy, or a total of four cycles are reached.

### 2.7. Nonresponders

No response is defined after 4 weeks of CC/Placebo CC administration and true acupuncture/control acupuncture with 3 consecutive weekly negative progesterone and hCG levels. Ovulation will be monitored by weekly serum progesterone level and ultrasound monitoring will not be performed as in a previous study [4]. Then during the fifth week a higher dose consisting of one more tablet per day (up to 3 tablets per day at third or fourth cycles, if they remain nonresponders) is given for 5 days, together with 2 weekly sessions of acupuncture. If there is no period after 3 weeks and the woman is not pregnant, CC 100 mg or 2 placebo tablets will be given daily from day 3 to day 7. If there is again no period after 3 weeks and the woman is not pregnant and not ovulating by progesterone levels, CC 150 mg or 3 placebo tablets will be given daily from day 3 to 7. The same CC dose will be continued in the next cycle if she is shown to have ovulation. A maximum of CC 150 mg or 3 placebo tablets daily are to be given even if she does not ovulate during the study period. Nonresponders will not receive progesterone for withdrawal bleeding before a higher dose of CC unless at discretion of the site investigator.

### 2.8. Pregnancy Visits

Serum progesterone and hCG levels are determined locally at sites to document ovulation and pregnancy during weekly visit for acupuncture treatment. Serial serum hCG level will be checked on a weekly basis if pregnant. Serum beta hCG > 10 IU/L is usually considered to be positive and indicates pregnancy. Pelvic ultrasound will be arranged to confirm the number and location of the gestational sac(s) when serum hCG is around 1500 IU/L or clinically indicated. Then pelvic ultrasound will be arranged every two weeks until viability of the pregnancy is determined by visualization of fetal heart motion. Pregnant subjects will be referred to the obstetrics unit and follow-up scans will be performed at week 18–24, 32, and 36 or at the discretion of the obstetrician.

We do not encourage the pregnant subjects to take any drugs during pregnancy. However, in Mainland China, pregnant women may be given many medications including TCM when they present with the risk of miscarriage. In this trial, pregnant women who presents with threatened miscarriage or are considered to have a significant risk for miscarriage can be given oral Duphaston (10 mg twice a day, Solvay Pharmaceuticals B.V.) till 12 weeks of gestation or 1 week after the vaginal bleeding stops as some studies [44–46] have shown benefit of Duphaston in threatened miscarriage. This information will be documented in the record form. No other medications including hCG, herbal medications, and acupuncture will be given by the investigators. 

### 2.9. End of Treatment Visit

At the end of study, a visit will be arranged for all women. The end of treatment visit should be performed as early as possible in pregnant women. A brief physical examination including acne and hirsutism assessment and a final assessment of adverse events and concomitant medications will be performed. Subjects will return the remaining study drug and their journal logs. All baseline measures will be repeated in all subjects. 

The blood samples done at the baseline visit and the end of the treatment visit will be shipped to the core laboratory for analysis. 

### 2.10. Establishment of a Pregnancy Registry

The outcomes of all pregnancies will be followed after delivery or termination of gestation. We will review pregnancy and birth records of the mother and of the fetus to establish neonatal morbidity and mortality and the presence of fetal anomalies. We will extract from these records concomitant medical and obstetrical conditions, exposure information on all other medical products used, including prescription products, over-the-counter products, dietary supplements, vaccines, and insertable or implantable medical devices. We will file individual case reports for all congenital anomalies, which will be reported as serious adverse events. The ectopic pregnancies will be identified by serial hCG monitoring and pelvic ultrasound examination. The doctors performing pelvic ultrasound are blinded to the treatment groups.

### 2.11. Sample Size Calculations

The overall live birth rate for women to receive CC is estimated to be 20% during the 4-month period according to Chinese Obstetrics and Gynecology (Second Edition). Due to lack of existing data on the effectiveness of acupuncture, we cannot rely on the literature for samples size determination. Instead we hypothesize that the order of effectiveness of the treatments is as follows:

 True acupuncture and CC > Control acupuncture and CC,
 control acupuncture and CC > True acupuncture and placebo CC,
 true acupuncture and placebo CC > Control acupuncture and placebo CC.

The respective live birth rates are projected with 10% difference as follows and the average rate among four arms is 15% (Table 3).

 Using a two-sided trend test and with an effective sample of 200 per group, our study is designed to have >80% power at the significance level of 0.015 (we have three primary hypotheses). The sample size has been inflated from 200 to 250/arm to allow for a dropout rate of 20%, totally 1000 cases for four arms. 

There have been multiple trials to study the effects of CC in PCOS women. The primary aim of this trial is not to reassess the efficacy of CC. The aim is to elucidate the additional efficacy of acupuncture in combination with CC, or acupuncture alone. Acupuncture is compared with CC because CC has been demonstrated to be superior to metformin [4]. The previous study design is used as a role model since it was the best executed and most cited trials in this area. Dr. Legro chairs the Steering Committee of this trial, and Dr. Zhang leads the DCC for another trial using CC in PCOS women. They have considered all related studies, although the power calculation involved inevitably some assumptions. The significance level of 0.015 is approximately 0.05/3 because in this study the overall significance level is controlled to 0.05 after correcting for the multiple comparisons involving three primary hypotheses.

### 2.12. Data Analysis

The primary outcome is the live birth rate. Secondary outcomes include (1) ovulation rate; (2) ongoing pregnancy rate at gestation 8–10 weeks; (3) multiple pregnancy rate; (4) miscarriage rate: loss of an intrauterine pregnancy before 20 completed weeks of gestation; (5) change in hormonal profile: FSH, LH, T, SHBG, and DHEAS; (6) change in metabolic profile: glucose and insulin concentrations, cholesterol, triglycerides, HDL-C, and LDL-C; (7) side effect profile; (8) change in QOL; (9) change in symptoms of anxiety and depression; and (10) credibility assessments.

The analysis will use an intent-to-treat (ITT) approach to examining differences in the live birth rates among the four treatment arms. Primary efficacy analysis will be done by comparing the treatment groups with respect to the primary outcome of live birth using the Pearson Chi-square test. For the secondary, supportive analysis, we will fit a logistic regression model to compare the treatment arms with respect to the primary outcome of live birth, adjusting for other factors such as randomization stratification of study site and prior exposure to study medications. The analysis of other secondary outcomes measured over time will entail the application of statistical methods that have been developed for correlated data since repeated observations will be made over time on each individual. For secondary outcomes such as hormone levels, a linear mixed-effects model will be fit where the main independent variables will be treatment group, time, and their interaction as well as the designed randomization stratification factors as covariates. Logistic regression models will be used in secondary analyses to evaluate the predictive value of treatment arm, clinical site, prior exposure to CC, body mass index, and other explanatory variables on binary outcomes (e.g., singleton live birth, abortion). Cox proportional hazards models and a Kaplan-Meier method will be applied to compare time to pregnancy in the treatment groups. 

Adverse events will be categorized and percentage of patients experiencing adverse events and serious adverse events in this trial will be documented. Chi-square tests will be performed to examine differences in the proportion of total and categories of adverse events within each treatment arm. For each Data and Safety Monitoring Board (DSMB) report, a list and summary of the reported adverse events will be presented in a blinded fashion, unless otherwise formally requested. Unblinding of treatments will take place after all participants have delivered or reported final outcomes to the DCC or when there are medical emergencies.

## 3. Results

### 3.1. Trial Registration and Conduct

The trial has been registered at clinicaltrials.gov (NCT01573858) as well as the Chinese clinical trial registration site (http://www.chictr.org/cn/; ChiCTR-TRC-12002081), prior to the start of randomization. The study protocol, the manual of operations and procedures (MOP), CRFs, and informed consent forms have been reviewed and approved by each site participating in the study. They are in both English and Chinese versions. The principal investigators (PIs) of the protocol and the grants supporting the study are Professor Xiaoke Wu and Professor Lihui Hou of Heilongjiang University of Chinese Medicine. Professor Heping Zhang at Yale University leads the DCC with the help of personnel from Heilongjiang University of Chinese Medicine. All study sites are monitored on a regular basis, the quality of data is tracked, and protocol deviations and violations are reported. 

### 3.2. Study Funding, Governance, and Data Sharing

The study is funded by National Public Welfare Projects for Chinese Medicine and National Trial Base for TCM and is led by a steering committee consisting of the two PIs, the leader of the DCC, an independently-selected chair (Professor Richard S. Legro of Penn State College of Medicine), site leaders and investigators, and other selected experts. Each of these individuals has one vote. Decisions are reached by majority consensus and a formal vote. The steering committee meets three times a year face to face and communicates through phone conferences or emails regularly. Additionally there are subcommittees including a protocol committee, a recruitment committee, and publication committee. 

There is an independent DSMB, which has conferences regularly via phone to review the conduct and results of the trial. The DSMB consists of both Chinese and American members, and reports are prepared in both Chinese and English. The DSMB reviews the protocol and study progress and makes recommendations during the course of this trial. For example, it reviews immediately any serious adverse events and adjudicates them. The DSMB also could recommend termination of the trial for a variety of reasons including poor recruitment, adverse effects of study medications, or a clear trend in one of the blinded treatment arms in live births exceeding our expectations. There are no formal stopping parameters for this trial. 

### 3.3. Study Progress and Recruitment

The targeted duration of recruitment will be 18 months and 25 hospitals at 21 sites are involved. In order to achieve our enrollment goal of 1,000 randomized subjects, each site must contribute 40–50 subjects, which amounts to about one recruited subject per site per week. There are a four-month treatment period and another nine-month period to follow up the pregnancy outcome. Therefore from the initial recruitment to the completion of the follow-up, a total of 31 months (2.5 years) will be required to complete the study. Up to the end of April 2013, 326 subjects were recruited.

## 4. Discussion 

 This clinical trial is to test the efficacy of acupuncture with or without CC on live birth in PCOS women suffering from ovulatory dysfunction. Previous clinical and experimental studies indicate that acupuncture may induce ovulation and improve pregnancy in these women. In the design of an acupuncture trial it is of great importance to define an adequate dose of acupuncture for the specific condition to be studied by taking all existing clinical and experimental findings into account [47], and to follow the STRICTA guidelines [29]. Further this clinical trial consists of a unique collaboration among investigators from China mainland, Hong Kong, Sweden, and the United States, with a similar China-US DSMB to oversee the trial. We have created study source documents in both Chinese and English and meetings and materials exist simultaneously in both languages. This international collaboration has presented us with unique opportunities (such as attracting expertise and resources from different international institutions) and challenges (e.g., coordinating activities in different time zones and training investigators who have limited experience in multicenter studies). As a result, in order to uphold the highest ethical standard and data quality, communication and repeated trainings have been extremely important. 

In this study, all acupuncture treatments are given by well-trained TCM doctors who received training in PCOS, theoretical and practical acupuncture for our protocol. This training included the reviews of previous clinical and experimental studies and discussions with clinicians and the steering committee members. Before the start of the study, we conducted a one-day study-specific education, including theoretical sessions with a Western Medical approach to acupuncture physiology, practical sessions with demonstrations of true and control acupuncture, and lectures on research methodology with focus on RCT. Periodic updates have been conducted on an annual basis. Importantly, the DCC personnel have been trained at Yale University Collaborative Center for Statistical Science for the RCT conduction (e.g., coordination, reporting, and data management), and clinical investigators have been trained at Harbin and Beijing.

Three control procedures are commonly used in acupuncture clinical trials: waiting-list, nonpenetrating needle, and needle-insertion (superficial or deep) controls. Waiting-list control was more frequently used in earlier RCTs. Nonpenetrating sham needle control, also known as “placebo control,” with a device that looks and feels like a real acupuncture needle but has a blunted tip that retracts into a hollow shaft handle and touches the skin without penetration [48] and needle-insertion control, with superficial needle placement at acupuncture points or at nonpoint or “wrong” point, or deep needle placement at “wrong points” [49, 50] are all frequently used control situations. Importantly, none of these control procedures are inert. As previously mentioned, the design of true acupuncture is based on existing data from experimental studies, on a previous RCT in PCOS women [20], and on an unpublished RCT registered at ClinTrial.gov (NCT00921492). 

The control acupuncture is chosen in contrast to true acupuncture and designed in such way that we believe it is least effective. The needles used in control acupuncture, which are thinner and shorter than those in true acupuncture, are inserted superficially in the skin without any stimulation, one in each shoulder and on in each upper arm at nonacupuncture points. Placement of needles in the control acupuncture is unlikely to induce ovulation in women with PCOS. The electrodes are attached to the needles but without any stimulation in order to mimic EA in the true acupuncture. The use of control acupuncture is not only to keep the study investigators (except the acupuncturists) blinded to the treatment but also to let the participants know that a certain acupuncture was performed to minimize the bias of the psychological effects receiving or not receiving a perceived effective acupuncture treatment. The participants are informed that two acupuncture protocols are tested, but they do not know which one is better in achieving ovulation and live birth. 

We have further created a unique study team consisting of experts in Western and TCM, clinical trials, biostatistics, and ethics, and developed a multilanguage infrastructure to conduct and monitor the trial. This model both serves to provide additional oversight and quality control to the trial but also allows for the mutual education of all participating investigators in areas where their previous training or experience was limited. 

## Figures and Tables

**Table 1:** Acupuncture points, stimulation, localization, tissue in which needles are inserted, and innervations area in true acupuncture which consists of two protocols that are alternated every other treatment (A and B) and control acupuncture.

True acupuncture point	Stimulation	Localisation	Muscle	Muscle innervation
*Protocol A *				
Zhongji; CV3	EA	4 cun caudal to the umbilicus	Fibrous tissue, linea alba	L1
Qihai; CV6		1.5 cun caudal to the umbilicus	Fibrous tissue, linea alba	Th11
Guilai; ST29	EA	1 cun cranial to the pubic bone and 2 cun lateral to the midline	M rectus abdominis	Th6-12
Sanyinjiao; SP6	EA	3 cun proximal to the medial malleolus	Mm. flexor digitorum longus, tibialis posterior	L4-5, S1-2
Yinlingquan; SP9		Below medial tibia condyle	M. gastrocnemius	S1-2
Hegu; LI4	DeQi 4 times	On the highest point at m. interosseus dorsalis	Mm. interosseus dorsalis I, lumbricalis II, adductor pollicis	C8, Th1
Baihui; GV20	DeQi 4 times	On the top of the head	Aponeurosis epicranii	C2-3, N. trigeminus

*Protocol B *				
Zhongji; CV3	DeQi 4 times	1 cun cranial to the pubic bone in the midline	Fibrous tissue, linea alba	L1
Qihai; CV6	DeQi 4 times	1.5 cun caudal to the umbilicus	Fibrous tissue, linea alba	Th11
Guilai; ST29	EA	1 cun cranial to the pubic bone and 2 cun lateral to the midline	M rectus abdominis	Th6–12
Tianshu; ST25		2 cun lateral to the midline at the level of the umbilicus	M rectus abdominis	Th6–12
Sanyinjiao; SP6	EA	3 cun proximal to the medial malleolus	Mm. flexor digitorum longus, tibialis posterior	L4-5, S1-2
Taichong; LR3		Between metatarsale I & II, just distal to the caput	M. Interosseus dorsalis I	S2-3
Neiguan; PC6	DeQi 4 times	2 cun proximal to processus styloideus radii, between the tendons of palmaris longus and flexor carpi radialis	M. flexor digitorum superficialis	C8, Th1
Baihui; GV20	DeQi 4 times	On the top of the head	Aponeurosis epicranii	C2-3, N. trigeminus

Control acupuncture point	Stimulation	Localisation	Skin	Skin innervation

No known point	Sham EA	On top of acromion	Skin	C3-4, n. supraclavicularis
No known point	Sham EA	On humerus, behind LI14	Skin	C5-6, n. cutaneous brachii lateralis

CV: conception vessel; ST: stomach; SP: spleen; LI: large intestine; GV: governor vessel; LR: liver; PC: pericardium.

**Table 2:** Overview of the study visits.

	Screening visit	Baseline visit	Week 1		Week 2		Week *N* + 1		Week 16		End of treatment visit
Visit #	1	2	3	4	5	6	*N*	*N* + 1	33	34	35

Sign consent	×										
History	×										×
Urine pregnancy test	×		×	×	×	×	×	×	×	×	
Physical examination	×										×
Transvaginal ultrasound	×										×
Semen analysis	×										
Hysterosalpingogram	×										
Safety eligibility lads	×										×
Fasting phlebotomy for study parameters		×									×
Depression, anxiety, and quality of life questionnaires	×										×
Credibility check questionnaire			×		×						×
Progesterone assay	×			×		×		×		×	×
HCG assay	×			×		×		×		×	×
Acupuncture treatments (twice weekly)			×	×	×	×	×	×	×	×	
Assess adverse events and concomitant meds				×		×		×		×	×

Credibility check questionnaires are completed on the third acupuncture treatment and the last acupuncture treatment.

HCG: HCG concentrations will be measured once a week during the study period. If serum HCG concentration is elevated above 10 IU/L, pelvic ultrasound will be arranged 2 weeks later for the number of the gestational sacs and the viability of the pregnancy.

**Table 3:** The respective live birth rates in each intervention.

Interventions	True acupuncture	Control acupuncture
CC	*A* = 25%	*B* = 15%
Placebo CC	*C* = 15%	*D* = 5%

## Abbreviations

ACE: Angiotensin-converting enzyme
CC: Clomiphene citrate
CNS: Central nervous system
CRF: Case report form
CV: Conception vessel
DCC: Data coordinating center
DHEAS: Dehydroepiandrosterone sulfate
DSMB: Data safety and monitoring board
E2: Estradiol
FSH: Follicle-stimulating hormone
GnRH: Gonadotropin releasing hormone
GV: Governor vessel
HDL-C: High-density lipoprotein cholesterol
HyCoSy: Hysterosalpingo contrast sonography
ITT: Intent-to-treat
LDL-C: Low-density lipoprotein cholesterol
LH: Luteinizing hormone
LI: Large intestine
LR: Liver
OCPs: Oral contraceptive pills
PC: Pericardium
PCOS: Polycystic ovary syndrome
RCT: Randomized controlled trial
SHBG: Sex hormone binding globulin
SP: Spleen
ST: Stomach
T: Total testosterone
TCM: Traditional Chinese Medicine.
